# Diabetes Mellitus Is Associated with Shortened Activated Partial Thromboplastin Time and Increased Fibrinogen Values

**DOI:** 10.1371/journal.pone.0016470

**Published:** 2011-01-28

**Authors:** Ying Zhao, Jie Zhang, Juanwen Zhang, Jianping Wu

**Affiliations:** Department of Laboratory Medicine, The First Affiliated Hospital, College of Medicine, Zhejiang University, Hangzhou, China; Universidad Peruana Cayetano Heredia, Peru

## Abstract

**Objective:**

This study was designed to examine the relationship between shortened activated partial thromboplastin time (APTT) and increased fibrinogen values with diabetes mellitus.

**Methods:**

APTT, prothrombin time (PT), fibrinogen, fasting plasma glucose (FPG) and glycosylated hemoglobin A1c (HbA1c) levels were measured in 1,300 patients. Patients were divided into three groups according to their HbA1c and FPG levels.

**Results:**

When participants were grouped according to their HbA1c levels, we found significantly shorter APTT values (26.9±5.6 s) and increased fibrinogen levels (3.1, 1.9–6.3 g/L) in the diabetes group when compared with the other two groups. When participants were grouped according to their FPG levels, we found significantly shorter APTT values (26.9±6.2 s) and increased fibrinogen levels (3.1, 1.8–6.2 g/L) in the diabetes group when compared with the euglycemic group.

**Conclusions:**

Shorter APTT and increased fibrinogen levels might be useful hemostatic markers in patients with diabetes and in patients at high risk for diabetes.

## Introduction

Patients with diabetes mellitus have a high risk of atherothrombotic events. Many studies have shown a variety of diabetes mellitus-related abnormalities in hemostasis and thrombosis [1], [2]. Venous thrombosis has also been found to occur more frequently in diabetics. Eighty percent of patients with diabetes mellitus die due to thrombosis, and 75% of these deaths are due to cardiovascular complications. The vascular endothelium is the primary site of defense against thrombosis and is abnormal in patients with diabetes mellitus [3].

Although modern coagulation diagnostic tests are becoming more sophisticated, standard coagulation screening tests, such as activated partial thromboplastin time (APTT) and prothrombin time (PT), are still important basic examinations in clinical laboratories. APTT is commonly used to test the intrinsic coagulation pathway, where a prolonged APTT is a clinical indicator of either a factor deficiency or the presence of coagulation inhibitors [4]. Recent studies have also shown that shortened APTTs may also reflect procoagulant imbalances with increased levels of coagulation factors. Therefore, APTT can be used to assess the risk of thromboembolic complications in patients with diabetes mellitus [5], [6].

Plasma fibrinogen levels influence thrombogenesis, blood rheology, blood viscosity and platelet aggregation. Epidemiological studies have found a significant association between fibrinogen levels and insulin levels [7], [8]. Markers of fibrinolysis are abnormal in people with metabolic syndrome, and fibrinolytic dysfunction is markedly increased in subjects with diabetes mellitus and abdominal obesity [7], [9]. In addition, chronic hyperglycemia and tissue glycation have marked effects on fibrin structure, clot generation and resistance to fibrinolysis [7].

In the past, the American Diabetes Association (ADA) did not recommend the use of HbA1c assays in the diagnosis of diabetes [10], principally because HbA1c assays were not standardized. HbA1c assays are now highly standardized and an international expert committee has recommended the use of HbA1c assays in the diagnosis of diabetes with a threshold of ≥6.5%[10]. The ADA has since concurred with this recommendation [10].

In the present study, we collected clinical data related to general coagulation function, as well as FPG and HbA1c levels from subjects during admission to hospital. Participants were divided into three groups based on either HbA1c or FPG levels [10]. The groups based on HbA1c levels were delineated as follows: normal group (HbA1c, ≤5.6%); high-risk diabetic group (HbA1c, 5.7% to 6.4%) and diabetic group (HbA1c, ≥6.5%). The groups based on FPG values were as follows: euglycemic group (FPG, <5.6 mmol/L), impaired fasting glucose group (IFG; FPG, 5.6 to 6.9 mmol/L) and diabetic group (FPG, ≥7.0 mmol/L). The purposes of the present study were to evaluate whether shortened APTTs and increased fibrinogen levels are related to increased FPG and HbA1c levels, and to assess the differences of APTT and fibrinogen levels among the three groups.

## Materials and Methods

### Patients

#### Inclusion criteria

The study included 1,300 patients (817 men and 483 women; median age, 64 yr; range, 39–83 yr) who were admitted to various clinical departments in the first affiliated hospital of Zhejiang University between May 2009 and February 2010. They all underwent APTT, PT, fibrinogen, FPG and HbA1c measurements. Medical diagnoses were obtained from the registered hospital records, including 726 patients with type 2 diabetes diagnosed according to the 1998 World Health Organization guidelines [11] (average duration of diabetes mellitus, 9.6 yr; range, 2∼25 yr) and 574 cases of patients with other diseases, except those listed in the exclusion criteria.

#### Exclusion criteria

Hypercoagulable states is broadly defined as encompassing two clinical situations: i) the presence of laboratory abnormalities, such as thrombocytosis or antithrombin III deficiency, or clinical conditions, such as cancer, pregnancy, or the postoperative state, that have been considered to be associated with an increased risk of thromboembolic complications (prethrombotic states); and ii) recurrent thrombosis in patients who have no recognizable predisposing factors (thrombosis-prone patients) [12]. Patients were excluded if they had a past history of a predisposition to hypercoagulability, including the following: thrombocytosis; a history of venous thromboembolism; known inherited coagulation disorders; cancer; pregnancy; recent surgery; hyperthyroidism; or patients who were taking standard anticoagulant treatment with either coumarin derivatives or heparins at the time of admission. Patients with type 1 diabetes and a body mass index (BMI) equal or greater than 28 kg/m^2^ [13] were also excluded from the study.

### Ethics statement

This study was approved by the ethics committee of the First Affiliated Hospital of Medical college at Zhejiang University, China, and was in accordance with the Helsinki declaration. Written informed consent was obtained from each of the participants at the time of enrollment.

### Assays

All specimens for APTT, PT, fibrinogen, FPG and HbA1c measurements were obtained by venepuncture in the morning after a 12 h fast. APTT, PT and fibrinogen levels were measured with the coagulation method on a Sysmex CA7000 System (Sysmex, Kobe, Japan) using Dade Behring reagents (Dade-Behring, Marburg, Germany). FPG was determined on an Abbott Aeroset (Abbott Laboratories, Illinois, USA) using Roche reagents (Roche Diagnostics, Indianapolis, USA). HbA1c was determined on a Bio-Rad Variant II HbA1c analyzer (Bio-Rad, California, USA). Our laboratory constructed our own reference ranges for APTT, PT and fibrinogen, according to the CLSI C28-A2 [14]. The reference ranges used were as follows: 22.0–36.0 s for APTT; 10.5–14.0 s for PT; and 2.0–4.0 g/L for fibrinogen. BMI as an index to estimate the extent of obesity was calculated by body weight (kg) divided by the square of the height (m^2^) [13].

### Statistics

Statistical analysis was carried out using SPSS, version 11.5. PT and APTT results were normally distributed and were reported as the mean ± standard deviation. Fibrinogen levels, age and BMI values were not normally distributed and were therefore reported as the median ±5^th^ -95^th^ percentile distribution. The significances of the differences in PT and APTT between groups were determined using a one way analysis of variance (ANOVA). The significance of differences of fibrinogen, age and BMI between groups were determined using the Mann-Whitney U test. The significance of differences in sex, shortened PT, APTT and increased fibrinogen between groups were compared with the Chi-square test. All statistical tests were 2-tailed with p-values <0.05 taken as significant.

## Results

The sex, age, BMI, PT, APTT and fibrinogen levels of the study participants were compared after they were grouped according to HbA1c or FPG levels (Tables 1 and 2). There were no significant differences in terms of sex, age and BMI differences among these two sets of three groups. When the high-risk diabetic group was compared with the normal group, statistically significant differences were observed in overall APTT (p = 0.049), APTT <22 s (p = 0.018) and fibrinogen levels (p = 0.036). When the diabetic group was compared with the normal group, statistically significant differences were observed in overall APTT (p<0.001), APTT <22 s (p = 0.001), PT (p = 0.016), PT <10.5 s (p = 0.012), fibrinogen levels (p<0.001) and fibrinogen >4.0 g/L (p = 0.004). When the diabetic group was compared with the high-risk diabetic group, statistically significant differences were observed in the overall APTT (p = 0.041), fibrinogen levels (p<0.001) and fibrinogen >4.0 g/L (p<0.001). We also found significantly shortened APTT values (26.9±5.6 s) and increased fibrinogen levels (3.1, 1.9–6.3 g/L) in the diabetic group than in the other two groups. APTT <22 s and fibrinogen >4.0 g/L were more frequently observed in the diabetic group (16.2% and 27.2%, respectively) than in the other two groups.

**Table 1:** APTT, PT and fibrinogen in patients grouped by HbA1c levels.

	HbA1c(%)		
	≤5.6	5.7–6.4	≥6.5
N	151	373	776
Age(years)	63(36–86)	63(42–83)	64(40–82)
Female/male	64/87	127/246	292/484
BMI(kg/m^2^)	22.8(19.8–27.2)	23.1(20.1–27.3)	23.3(20.2–27.4)
APTT(s)	28.8±7.0	27.6±6.5*	26.9±5.6*△
APTT<22 s	9(6.0%)	49(13.3%)*	126(16.2%)*
PT(s)	11.7±1.3	11.5±1.4	11.4±1.5*
PT<10.5 s	13(8.6%)	57(15.3%)	163(21.0%)*
Fibrinogen(g/L)	2.7(1.6–5.3)	2.9(1.7–5.9)*	3.1(1.9–6.3)*△
Fibrinogen>4.0 g/L	24(15.9%)	63(16.9%)	211(27.2%)*△

*p<0.05 comparison with HbA1c≤5.6%;△ p<0.05 comparison with HbA1c 5.7–6.4%.

**Table 2:** APTT, PT and fibrinogen in patients grouped by FPG levels.

	FPG(mmol/L)		
	<5.6	5.6–6.9	≥7.0
N	590	279	431
Age(years)	64(39–84)	63(39–83)	63(33–79)
Female/male	217/373	110/169	156/275
BMI(kg/m^2^)	23.0(20.3–27.2)	23.3(19.8–27.2)	23.3(20.2–27.5)
APTT(s)	27.8±6.2	26.8±5.5*	26.9±6.2*
APTT<22 s	67(11.4%)	40(14.3%)	77(17.9%)*
PT(s)	11.5±1.4	11.4±1.3	11.4±1.5
PT<10.5 s	84(14.2%)	48(17.2%)	101(23.4%)*△
Fibrinogen(g/L)	3.0(1.8–5.9)	3.1(1.8–6.3)	3.1(1.8–6.2)*
Fibrinogen>4.0 g/L	117(19.8%)	68(24.3%)	113(26.2%)*

*p<0.05 comparison with FPG<5.6 mmol/L;△ p<0.05 comparison with FPG5.6–6.9 mmol/L.

In terms of the three groups divided according to their FPG values [10], when the IFG group was compared with the euglycemic group, a statistically significant difference in overall APTT (p = 0.034) was observed. When the diabetic group was compared with the euglycemic group, significant differences were observed in overall APTT (p = 0.031), APTT <22 s (p = 0.003), PT <10.5 s (p<0.001), fibrinogen levels (p = 0.002) and fibrinogen >4.0 g/L (p = 0.016). When the diabetic group was compared with the IFG group, significant differences were noted only in PT <10.5 s (p = 0.046).

## Discussion

Patients are considered to have a hypercoagulable state if they have laboratory abnormalities or clinical conditions that are associated with increased risk of thrombosis; diabetic patients meet these criteria [12], [15]. Hyperglycemia contributes to the hyperfibrinogenemia of diabetic patients and activates the coagulative cascade, thus increasing thrombin formation and fibrinogen degradation products, which may stimulate hepatic fibrinogen synthesis [12], [16]. Diabetic patients have elevated levels of fibrinogen and factors in the intrinsic pathway, which are determinants of APTT [6].

PT and APTT tests are standard screening tests for function of the coagulation system and their utility in monitoring therapeutic anticoagulation is widely accepted [17]. The APTT assay is traditionally used for identifying abnormalities in the contact (factor XII, prekallikrein, and high-molecular-weight kininogen), intrinsic (factors XI, VIII, IX) and common (factors X, V and II and fibrinogen) pathways of coagulation [5]. Prolonged APTT values have clinical relevance as an indicator of factor deficiency or the presence of coagulation inhibitors [4]. Shortened APTTs are generally considered to be laboratory artifacts arising from problematic venepunctures[18]. However, there is mounting evidence that shortened APTT values in some cases may reflect a hypercoaguable state, which is potentially associated with increased thrombotic risk and adverse cardiovascular events [4], [6], [16]. Shortened APTTs may result from an accumulation of circulating activated coagulation factors in plasma caused by enhanced coagulation activation in vivo [6], [19].

The prothrombin time (PT) is the screening test for the coagulation pathway initiated by tissue factor. PT is most sensitive to factor VII (FVII) levels. Factor VII coagulant activity (FVII:c) levels were higher in the type 2 diabetes patients and metabolic syndrome individuals [7]. The activation of prothrombin occurs on the platelet surface in vivo, the addition of platelets to plasma and the activation of platelets accelerates the production of thrombin [15]. When thrombin is formed from prothrombin, prothrombin activation fragments 1 + 2 are released and these levels are increased in diabetes [20].

Increased fibrinogen levels are a strong and independent cardiovascular risk factor [16], [20]. In a hyperglycemic environment, fibrinogen can become hyperglycosylated [15]. When this abnormal fibrinogen clots, the resulting fibrin structure is composed of small diameter fibers that are markedly resistant to degradation by plasmin [15].

Shortened APTTs values are found with a prevalence of 2.5%–5% in hospitalized patients [8], [17]. In our study, whether patients were grouped according to HbA1c or FPG levels, the APTT values in the diabetic, high-risk diabetic or IFG groups were significantly shorter than in the normal groups, and APTT values below the reference range (APTT <22 s) were more frequent. Additionally, fibrinogen levels were significantly higher in the diabetic and high-risk diabetic groups than in the normal group, and fibrinogen values above the reference range (fibrinogen >4.0 g/L) were more frequent in the diabetic groups. Therefore, the results of this study clearly show that diabetic and high-risk diabetic patients have shortened APTTs and elevated fibrinogen levels.

Lippi et al. [6] showed that IFG and diabetic groups had significantly shortened APTT values and suggested that APTT might identify diabetic patients at major risk of thrombosis. In contrast, in our study, when patients were grouped by their HbA1c levels, the APTT values of the diabetic group were significantly shorter than the high risk group (Table 1); furthermore, when patients were grouped according to FPG levels, the APTT and fibrinogen values in patients with diabetes were not significantly different than the IFG group (Table 2). The difference in the two studies may be due to a lack of concordance between the HbA1c and FPG tests. Analyses of data from the National Health and Nutrition Examination Survey (NHANES) indicate that the HbA1c threshold of ≥6.5% identifies one-third fewer cases of undiagnosed diabetes than a fasting glucose cut-off point of ≥7.0 mmol/l [10], which results in differences in study populations depending on the diagnosing tests. Additionally, HbA1c levels incorporate contributions from both fasting and post-prandial glucose, which suggests that it gives a better representation of glucose intolerance as a whole. HbA1c has a higher reproducibility rate than FPG and has less intra-individual variation [22]. Avignon et al. [23] recently demonstrated that postprandial glucose concentrations correlated independently and significantly with HbA1c in type 2 diabetic individuals, whereas fasting plasma glucose levels did not. The variability of HbA1c values is also considerably less than that of FPG levels, with day-to-day within-person variance of <2% for HbA1c but 12–15% for FPG [22].

Fibrinogen levels have been shown to be elevated in type 2 diabetes mellitus patients and to predict the development of type 2 diabetes in healthy individuals [24]. A positive correlation between plasma glucose and fibrinogen levels has been reported in large epidemiological studies [25]. In 1990, Van Wersch et al. [26] observed increased fibrinogen concentrations in diabetic patients but no significant difference in comparison with the reference group. Similarly to the results of Lippi et al. [6] and Rotterdam et al. [27].Acang and Jalil [28] observed that there were significantly higher fibrinogen levels, and shortened PT and APTT values, in diabetic patients, especially in patients with long-term diabetes with chronic complications, which are consistent with the results of this study.

Madi et al. [21] provided compelling preliminary evidence that a shortened APTT had potential value for the early diagnosis of myocardial infarction among patients hospitalized for chest pain. Korte et al. [29] also found that patients presenting with shortened APTT values had increased thrombin generation, were in a complex hypercoagulant state and were at increased risk for thromboembolism. Tripodi et al. [5] found that hypercoagulability detected by shortened APTT values was independently associated with venous thromboembolism (VTE) and hypothesized that shortened APTT could be considered as a risk marker for VTE.

While the clinical relationship between shortened APTT, increased fibrinogen levels and the risk of venous thrombosis is supported by current scientific literature, the exact biological mechanisms of thrombosis in diabetics are likely to be multifactorial and incompletely understood as yet [6], [30]. Many authors have suggested that hemorheological disturbance is an important factor in the development of vascular complications. Alao et al. [31] showed elevated levels of total cholesterol, high density lipoprotein (HDL), low density lipoprotein (LDL) and triglycerides in patients with diabetes mellitus compared to non-diabetic controls; in particular, elevated LDL was a major risk factor for coronary artery thrombosis, which confirmed that patients with diabetes mellitus were predisposed to dyslipidemia, with higher risks for vascular disorders. The hypercoagulable state has also been suggested as a potential risk factor [28].

Endothelial abnormalities play a critical role in the enhanced activation of platelets and clotting factors that occur in diabetic patients. In such patients, coagulation activation markers are elevated and coagulation abnormalities seem to be directly linked to hyperglycemia, involving all stages of coagulation. Chronic hyperglycemia results in hyperglycosylation of multiple proteins [15]. The plasma levels of many clotting factors, including fibrinogen, factor VII, factor VIII, factor XI, factor XII, kallikrein, and von Willebrand factor are elevated in diabetes [6], [15].

In summary, clinical tests for APTT and fibrinogen are relatively inexpensive and are readily available. The results shown in this study indicate that shortened APTT and increased fibrinogen levels might be useful hemostatic markers in diabetic patients, especially in those at high-risk for thrombotic complications. Further investigations of these markers could potentially be used as screening tests for hypo- and hypercoaguable states that are applicable to diabetes and other clinical conditions.
